# Assessing the medium-term effects of a community-based medical education program with homestay practice: a cohort study in Tamba, Japan

**DOI:** 10.1080/07853890.2024.2396560

**Published:** 2024-08-29

**Authors:** Tsuneaki Kenzaka, Shinsuke Yahata, Ken Goda, Ayako Kumabe, Nishisaki Hogara, Masanobu Okayama

**Affiliations:** a Division of Community Medicine and Career Development, Kobe University Graduate School of Medicine, Kobe, Japan; b Department of Internal Medicine, Hyogo Prefectural Tamba Medical Center, Tamba, Japan; c Division of Community Medicine and Medical Education, Kobe University Graduate School of Medicine, Kobe, Japan

**Keywords:** Community medicine, community-based medical education, homestay, residency program, medium-term effect, attitude, rural area

## Abstract

**Introduction:**

This study intended to evaluate the medium-term effectiveness of a community-based medical education (CBME) program and to determine the program’s influence on the application rates of regional-quota students seeking to become residents in Tamba, Japan.

**Materials and Methods:**

We conducted a cohort study of regional-quota students. Exposure factors included (1) experience compared to no experience of CBME in the Tamba area; (2) CBME experience compared to no experience in Tamba in the senior years (4–6 years of medical school) and experience in the junior years (1–3 years of medical school); and (3) experience in the senior years compared with those in the junior years. Outcome measures were applications to become a medical resident and actually becoming a medical resident at the Hyogo Prefectural Tamba Medical Center.

**Results:**

Of 94 participants, 58 (61.7%) were male and 37 students (39.4%) had previous CBME experience in the Tamba area. In applying to become a resident at the Hyogo Prefectural Tamba Medical Center, students who had experienced CBME in the Tamba area in their senior years had significantly higher adjusted risk ratios compared to those who experienced it in their junior years. Regarding applications to become a resident, students who had experienced CBME in the Tamba area in their senior years had a significantly higher adjusted risk ratio than students who had not experienced CBME and students who had experienced CBME in their junior years.

**Conclusions:**

There was a statistically significant application rate for residency programs among medical students who participated in the CBME program in their senior years compared with those who did not. This is the first study to confirm the medium-term effects of CBME after several years in short-term CBME programs of three days and two nights.

## KEY MESSAGES

Medical students who participated in our community-based medical education (CBME) program in their senior year were statistically and significantly more likely to apply for residency programs than those who did not.

A short-term CBME program of three days and two nights incorporating a homestay practicum showed a medium-term effect after several years.

Close contact with local residents is likely to influence future work location selection.

## Introduction

Community-based medical education (CBME), a style of education in which medical students are immersed in the community, is becoming popular worldwide. CBME provides medical students with a broad range of skills and ethical competencies [1–3] and promotes their career intentions in primary care [1, 2] and community medicine, including rural healthcare [4, 5]. Rapid population aging is a major global healthcare issue [6]. To address this issue, more attention is being given to training healthcare professionals to provide primary and community medical care [7, 8]. It is important that such care be provided to communities in each region, including rural areas. These social contexts underscore the importance of CBME.

Several studies have demonstrated the medium- and long-term educational benefits of CBME programs [9–11]. These programs receive considerable financial support and involve long-term student stays and practical training in rural areas. They take place primarily in rural areas in various countries, where medical students participate in a carefully designed longitudinal integrated clerkship for an extended period of 6–54 weeks. Moreover, these CBME programs are designed to bring value to the community, including evidence-based practices and community health assessments [9]. Importantly, these programs are expected to significantly increase the number of medical students working in rural areas in the future.

CBME has been developing and spreading in Japan [12]. Japan’s Model Core Curriculum for Medical Education was developed in 2001, while the CBME section was first introduced in a revised version published in 2007 [13]. However, CBME in Japan with a specific focus on rural areas is less likely to receive financial support. Thus, CBME programs in rural Japan tend to be short-term stays of one day to two weeks at student training institutions [14–16]. Surveys conducted immediately after the placement showed a significant increase in medical students’ interest, fulfilment, and enthusiasm for rural medicine [14–16]. However, no results have been obtained for these short-term CBME programs in Japan showing medium-term practical training effects on an annual basis. To our knowledge, such short-term CBME programs have not yielded results showing effective mid-term practice effects worldwide.

When opportunities are provided to medical students to interact with local residents through community health assessments, they can learn about local residents’ lives and lifestyles. This contributes to the development of healthcare professionals responsible for primary care and community medicine [14, 17]. We conducted a CBME program in the Tamba area, Hyogo Prefecture, as a community medicine summer seminar. This CBME program was a three-day/two-night program that included a two-day/one-night homestay practice in a local resident’s house [18]. Close contact between students and local residents over extended periods of time, such as in a homestay practice, has the potential to enhance the effectiveness of CBME programs.

Indeed, as a short-term effect, incorporating this homestay practice in our CBME program significantly increased the number of students who chose it as their future workplace immediately after the practice [17]. However, the medium-term effect of this CBME on the actual choice of workplaces remains unknown. Longer-term CBME programs of 6–54 weeks have been shown to have medium- to long-term effects on actual workplace choice for medical students. However, a short-term CBME program of 1–2 weeks has not shown a medium-term effect on actual workplace choice. In our study too, the medium-term effects of our CBME program on actual workplace choice have thus far been unknown. Although our practical training is a short-term CBME program, it allocates significant time for close contact between students and local residents and may, therefore, have a medium-term effect on actual workplace choice. To determine the medium-term effectiveness of our CBME program, which includes a homestay program in the homes of local residents, we aimed to evaluate the effect of the CBME program on the application to become a resident or have a residency in Tamba, Japan, between regional-quota students.

## Materials and methods

This was a cohort study. All participants were regional-quota students in Hyogo Prefecture who graduated from medical school and started their initial training as part of a residency program between 2017 and 2021. The study was approved by the Ethics Committee of the Hyogo Prefectural Tamba Medical Center (Approval Number: Tan-I number 1061). All participants provided informed consent before participation.

### Background of Japanese medical education system and regional-quota students

#### The Japanese medical education system

In Japan, medical students receive six years of medical school education. Generally, from the fourth year onward, they undergo clinical clerkship training. After graduation from medical school, students undertake two years of initial resident training which is provided primarily at core hospitals. The choice of initial training hospital is made in the middle term of the sixth year of medical school. This is decided by matching medical students’ preferences for hospitals at which they want to do their initial training with the training hospitals that are willing to accept them. This matching is carried out by a third-party organization, the Japan Residency Matching Council.

#### University admissions selection of regional-quota students

Regional-quota students are determined by the University Entrance Examination Selection Test. In addition to the general academic examination and interview, regional-quota students are subjected to a specialized interview. The interview is conducted by an interviewer appointed by Hyogo Prefecture. Most medical schools in Japan have an admission capacity of approximately 100 students, in addition to which regional-quota students are admitted. Every year, Hyogo Prefecture admits 2–3 regional-quota students at Jichi Medical University, 5 at Hyogo College of Medicine, 10 at Kobe University, 0–2 at Tottori University, and 0–2 at Okayama University.

#### Difference in the curricula/teaching method between the universities

Regardless of whether they are regional-quota students, medical students at each university follow the same curriculum. Jichi Medical University specializes in rural medicine. Thus, its curriculum includes more hours of teaching and practical training in rural medicine than the other four universities. There is no significant difference in curriculum and teaching methods between the other four universities. Regional-quota students in Hyogo are provided with common lectures and practical training specializing in rural medicine by Hyogo several times a year, regardless of the university they are enrolled in.

### Study design and setting

The CBME program in the community medicine summer seminar was offered by the Hyogo Prefectural Tamba Medical Center (Hyogo Prefectural Kashiwa Hospital until July 2019), located in the Tamba area, a rural area in Hyogo Prefecture, Japan. The Tamba area comprises the cities of Tamba and Sasayama. Tamba City has a population of approximately 60,000 and an area of 493.21 km^2^. Sasayama City has a population of approximately 40,000 and an area of 377.59 km^2^. This CBME program ran for three days and two nights every August from 2015 to 2019; the local program has not been held since 2020 owing to the spread of coronavirus disease 2019 (COVID-19).

The educational program included off-the-job training on blood collection techniques and abdominal echoes using a simulator, mock interviews with local residents, and lectures provided by medical students to health residents. Students gained experience in local industries such as catching eelgrass fish and agriculture (planting vegetable seedlings). Subsequently, they visited important local historical sites. Students were also offered homestay programs through which they could stay in residents’ homes for one or two days and nights, allowing them to have close contact with local residents.

### Participants

The participants were regional-quota students who planned to work in the Hyogo Prefecture in the future. These students received a six-year medical student scholarship from Hyogo Prefecture and were exempt from repaying if they became physicians and worked in the rural area of Hyogo Prefecture for nine years. Approximately 70–80% of the regional-quota students enrolled as medical students attended the CBME program (the community medicine summer seminar) each year; their participation in the program was voluntary and non-participation did not affect their grades or promotion.

**Figure 1.**
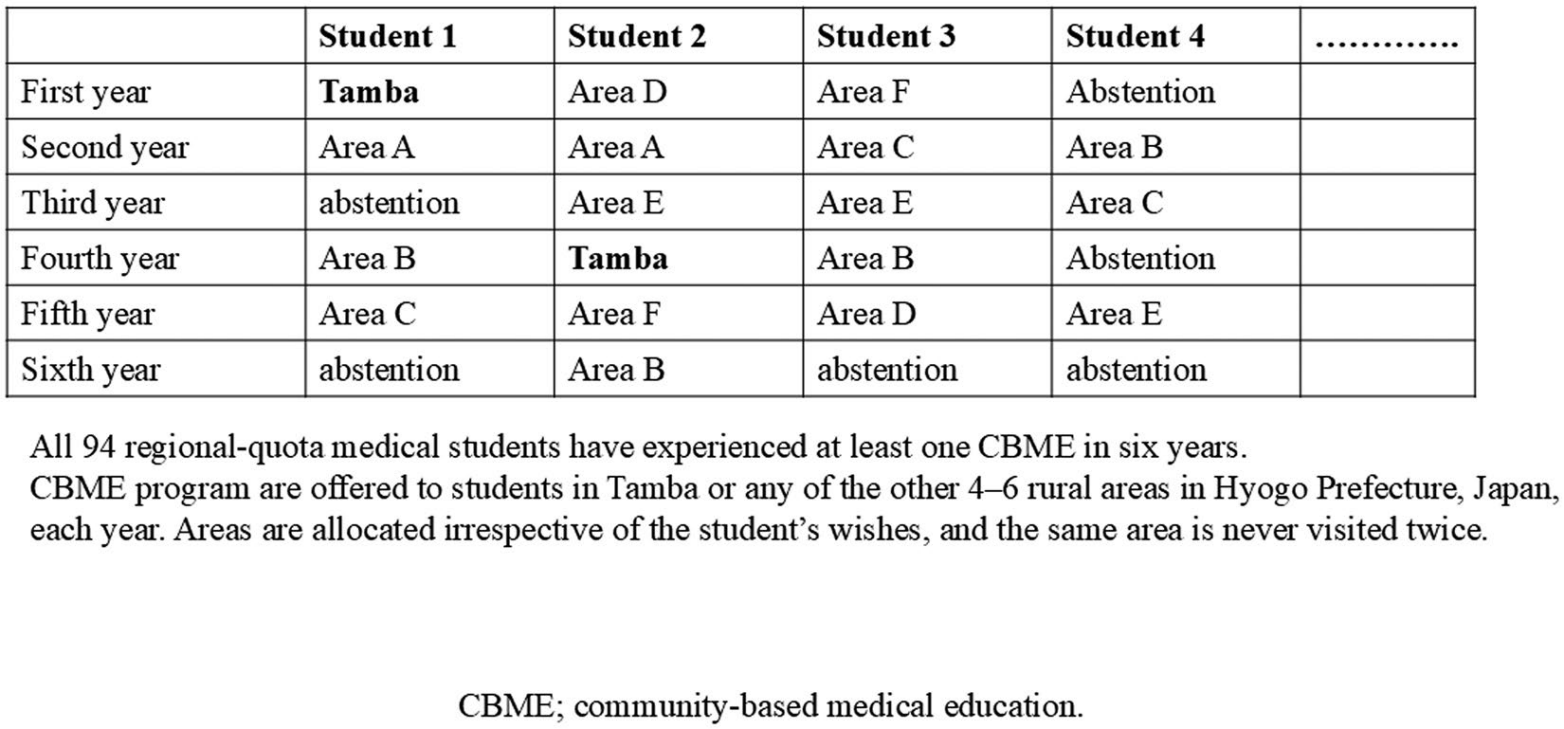
Diagram of where all regional-quota medical students experienced CBME for each academic year.

Each year, 10–15 regional-quota students were assigned to the Tamba area program. Other students received CBME in 4–6 rural areas in Hyogo Prefecture, outside Tamba. The assignments were performed randomly. Once a student was assigned to an area, they were unable to return to the same area to attend the CBME program in the following year. This is because CBME is a good opportunity for regional-quota students to learn about areas wherein they may have opportunities to work in the future, and therefore, they are required to become familiar with different areas. Figure 1 illustrates the distribution of regional-quota medical students’ participation in CBME across academic years.

The regional-quota medical students who participated in this CBME program from 2015 to 2019 would start their initial training as part of a residency program between 2017 and 2021. Therefore, the target population of this study is regional-quota students starting their initial training between 2017 and 2021 as doctors.

### Theoretical framework

The foundational theory of sense of community or psychological sense of community was proposed by Sarason in 1974 [19]. This concept was further developed by Macmillan and Chavis [20] who proposed a multidimensional model described as having ‘a sense of belonging, a sense that members matter to each other, and a shared belief that members’ needs will be met through a commitment to be together’. They proposed the following four-factor model, which they defined as: (1) membership, (2) influence, (3) integration and fulfilment of needs, and (4) shared emotional connection.

The theoretical construct of our study relates to the concept of medical students building a sense of community by being in close contact with local residents for a period of time.

It has already been reported that community health practice over a longer period of 6–54 weeks could be a factor in increasing the number of future medical students working in rural areas due to a sense of belonging and attachment to the community [9–11].

Our study verifies that even a short-term placement of three days and two nights may be a factor in increasing the number of future medical students working in rural areas due to a robustly built sense of community.

### Study materials

CBME in Hyogo Prefecture was offered to participants while they attended medical school. The CBME program was held simultaneously every summer at five to seven locations in the prefecture (Tamba area and four to six other rural areas). Practice sites for CBME were randomly selected, and no two visits to the same practice site were conducted. Off-the-job training, health lectures, social events with meals for residents, and visits to important local historical sites were offered in most CBME programs. Only in the Tamba area was a practicum, such as a homestay, offered. Homestays allow students to spend extended periods in close contact with local residents. All regional-quota medical students experienced the CBME program several times during their six years of study in one of the rural areas.

### Measurement tools

A cohort study was conducted with regional-quota medical students. The following demographic data were gathered from students: gender, university of origin, and whether they had practical training experience in the Tamba area (Hyogo Prefectural Tamba Medical Center). If students had experience in the Tamba area, the school year in which they had experienced the training was also recorded. The presence or absence of aspirations to become general practitioners was also assessed. We investigated whether they had applied to complete their initial training at Hyogo Prefectural Tamba Medical Center and whether they had actually become a resident there.

Exposure factors included: (1) experience compared with no experience of CBME in the Tamba area; (2) experience in the senior years (4–6 years of medical school) compared with experience in the junior years (1–3 years of medical school); and (3) experience of CBME in the Tamba area in the senior years compared with no experience of CBME and experience in the junior years.

The junior years (1–3 years of medical school) are in the Preclinical Phase. The senior years (4–6 years of medical school) are in the Clinical Phase.

The outcome measures were application to become a medical resident and becoming a medical resident at Hyogo Prefectural Tamba Medical Center.

Regional-quota students may work at any of the ten training hospitals in Hyogo Prefecture as doctors. They indicate their preferred hospital when they are in their sixth year of medical school. Hyogo Prefecture decides where they will work for their initial training based on the wishes of the regional-quota students.

### Data collection procedures

First, based on the list of participants of past community medicine summer seminars in the Tamba area (Hyogo Prefectural Tamba Medical Center) for the period 2015–2019, we investigated whether individual students received CBME training in the Tamba area among all regional-quota students.

Second, based on information from Hyogo Prefecture, we investigated whether individual students applied for initial training in the Tamba area and whether they actually trained there as an initial resident. The following data were obtained from internal program documents: regional-quota students’ gender, the university they graduated from, their desired medical department, their application to become a medical resident at Hyogo Prefectural Tamba Medical Center, and whether they actual became a medical resident there. These documents are managed by the Hyogo Prefecture, which is responsible for their accuracy. The data in them is provided by the regional-quota students and these documents officially determine the location to which they are assigned. In addition, these documents may be used for educational purposes, understanding career paths, and research purposes. All the regional-quota students consent to the data being used for these purposes. Nonetheless, we informed all the regional-quota students about this research and obtained their consent for the research through an opt-out method distributed through a mailing list.

### Data analysis

Participants’ demographic data were tabulated. This includes gender, university of origin, whether they wanted to be a general practitioner at the time of the training sites preferences survey, whether they applied to become a resident at the Hyogo Prefectural Tamba Medical Center, and whether they actually became a resident there. For ‘applying to becoming a resident in Tamba’ or ‘becoming a resident in Tamba’, risk ratios and 95% confidence intervals were analyzed for CBME experience in Tamba (with reference to none), male with reference to female, each university with reference to Jichi Medical University, and general practice intention (with reference to none). Their gender, university of origin, and whether the participants had aspirations to be a general practitioner at the time of the training site preference survey were treated as confounding factors. Risk ratios and 95% confidence intervals for each of the exposure factors were analyzed using Poisson regression with a robust error variance [21]. We analyzed the outcome variables ‘applying to becoming a resident in Tamba’ or ‘becoming a resident in Tamba’ separately. Risk ratios with a 95% confidence interval not including 0 were judged to be statistically significant. As this was a cohort study, Poisson regression analysis was used to calculate the risk ratios. The usual Poisson regression requires the assumption that the event occurrence is rare when subjects are followed for various lengths of time. To overcome this problem, this study adopted a modified Poisson regression analysis, which uses a robust error variance procedure. All statistical analyses were performed at Stanford University Medical Center using Stata MP, version 15 (StataCorp, College Station, TX, USA). As this was a pilot study and a cohort study of all regional-quota students, we did not consider the sample size.

## Results

### Basic demographic characteristics

Table 1 presents the basic demographic characteristics of the participants. These include gender, university of origin, whether they wanted to be a general practitioner at the time of matching, whether they applied to become a resident at the Hyogo Prefectural Tamba Medical Center, and whether they became a resident there. Of the 94 participants, 58 (61.7%) were male and 36 (38.3%) were female. Thirty-seven students (39.4%) had previous CBME experience in the Tamba area. Of these, 15 students had practical experience in their senior years, 18 (48.6%) applied to become residents in Tamba, and 8 (21.6%) actually became residents. Among the 57 students who had no CBME experience in Tamba, 27 (47.3%) applied for the residency program in Tamba and 9 (15.8%) actually became residents. In the basic participant demographics item, comparisons of ‘applying to become a resident in Tamba’ (Table 2) and ‘becoming a resident in Tamba’ (Table 3) showed no significant differences in gender or general practice intention. There were no significant differences among the universities; only Hyogo College of Medicine had significantly lower levels of applying to become a resident in Tamba, however, there were no significant differences elsewhere.

**Table 1.** Basic participant demographics.

	All	Experience	Control
	*n* = 94	*n* = 37	*n* = 57
	n (%)	n (%)	n (%)
Gender (male)	58 (61.7)	22 (59.5)	36 (63.2)
University			
Jichi Medical University	12 (12.8)	6 (16.2)	6 (10.5)
Hyogo College of Medicine	21 (22.3)	9 (24.3)	12 (21.1)
Kobe University	45 (47.9)	20 (54.1)	25 (43.9)
Tottori University	10 (10.6)	1 (2.7)	9 (15.8)
Okayama University	6 (6.4)	1 (2.7)	5 (8.8)
General practice intention*	31 (34.4)	12 (33.3)	19 (35.2)
Applying to become a resident in Tamba	45 (47.9)	18 (48.7)	27 (47.4)
Becoming a resident in Tamba	17 (18.1)	8 (21.6)	9 (15.8)

* Four participants’ data were missing.

**Table 2.** Comparison of applying to become a resident in Tamba in basic participant demographics.

	RR (95% CI)
CBME experience in Tamba	1.16 (0.74 to 1.83)
Male with reference to female	0.89 (0.59 to 1.33)
University (reference to Jichi Medical University)	
Hyogo College of Medicine	0.08 (0.01 to 0.59)
Kobe University	0.81 (0.51 to 1.29)
Tottori University	1.17 (0.65 to 2.12)
Okayama University	0.49 (0.14 to 1.71)
General practice intention at final school year	1.38 (0.14 to 1.70)

Abbreviations: CBME, Community-based medical education; CI, confidence interval; RR, risk ratio.

**Table 3.** Comparison of becoming a resident in Tamba in basic participant demographics.

	RR (95% CI)
CBME experience in Tamba	1.26 (0.53 to 2.96)
Male with reference to female	0.75 (0.31 to 1.79)
University (reference to Jichi Medical University)	
Hyogo College of Medicine	–
Kobe University	1.79 (0.42 to 7.66)
Tottori University	–
Okayama University	0.49 (0.14 to 1.71)
General practice intention at final school year	2.01 (0.89 to 4.56)

Abbreviations: CBME, Community-based medical education; CI, confidence interval; RR, risk ratio.

### Comparison of students with and without CBME experience in the Tamba area

Table 4 presents the risk ratios for students with and without CBME experience in the Tamba area. Risk ratios and 95% confidence intervals were analyzed using CBME experienced participants compared to CBME inexperienced participants in the Tamba area as exposure factors. Poisson regression with robust error variance was used in the analysis, treating gender, university of origin, and whether the patient wanted to be a general practitioner as confounding factors.

**Table 4.** Influence of an undergraduate community-based medical education (CBME) program in Tamba: risk ratios for students with CBME experience in Tamba compared to those without.

	RR (95% CI)	Adjusted RR* (95% CI)
Applying to become a resident in Tamba	1.03 (0.67 to 1.58)	1.17 (0.74 to 1.84)
Becoming a resident in Tamba	1.37 (0.58 to 3.23)	1.26 (0.54 to 2.97)

Abbreviations: CI, confidence interval; RR, risk ratio.

* Adjusted for gender, university, and general practice intentions in the final school year.

There were no significant results for the risk ratios for applying to the residency program or actually becoming a resident.

### Comparison between students with CBME experience in the senior and junior years

If a student experiences CBME in the first year of medical school, the choice of initial training hospital is made five years later, in the sixth year. In addition, clinical practice experience and medical knowledge are insufficient in the junior years (1–3 years of medical school). Thus, exposure factors were used to compare students who experienced CBME in their senior years with those who experienced it in their junior years among community-based students to ascertain how long the long-term effects of the practice would persist.

Table 5 presents the risk ratios of students with CBME experience in the Tamba area in the senior years compared with those with CBME experience in the junior years. Poisson regression with robust error variance was used in the analysis, treating gender, university of origin, and whether the participant wanted to be a general practitioner as confounding factors. The adjusted risk ratio was 2.23 (95% confidence interval 1.15 to 4.33), indicating that students who experienced the program in the senior years were significantly more likely to apply for the residency program in Tamba.

**Table 5.** Influence of an undergraduate community-based medical education (CBME) program in Tamba on students in their fourth year and above (restricted to CBME participants): risk ratios for students with CBME experience in the Tamba area in the senior years (4–6 years of medical school) compared to those of students with CBME experience in the junior years (1–3 years of medical school).

	RR (95% CI)	Adjusted RR* (95% CI)
Applying to become a resident in Tamba	2.30 (1.16 to 4.57)	2.23 (1.15 to 4.33)
Becoming a resident in Tamba	1.47 (0.43 to 4.97)	3.13 (0.55 to 17.90)

Abbreviations: CI, confidence interval; RR, risk ratio.

* Adjusted for gender, university, and general practice intentions in the final school year.

The effect of experiencing CBME in the senior years was shown to be statistically significant and to persist for several years (1–3 years).

### Comparison of students with CBME experience in the senior years, junior years, and no experience

As described above, students who experienced CBME in the senior years were statistically significantly more likely to apply to become a resident at Hyogo Prefectural Tamba Medical Center than those who experienced CBME in the junior years. Therefore, we considered students who had experienced CBME in their senior years to be an important exposure factor. Students who experienced CBME in the senior years were then compared as an exposure factor with students who did not experience CBME in the Tamba area and with students who experienced CBME in the junior years (Table 6 presents the risk ratios). Poisson regression with robust error variance was used in the analysis, treating gender, university of origin, and whether the patient wanted to be a general practitioner as confounding factors. The adjusted risk ratio was 1.58 (95% confidence interval 1.04 to 2.39), indicating that students who experienced the program in the senior years were significantly more likely to apply for the residency program in Tamba.

**Table 6.** Influence of an undergraduate community-based medical education (CBME) program in Tamba on students in their fourth year and above: risk ratios for students who experienced CBME in the senior years (4–6 years of medical school) compared to those of students with no CBME experience in the Tamba area and of students who experienced CBME in the junior years (1–3 years of medical school).

	RR (95% CI)	Adjusted RR* (95% CI)
Applying to become a resident in Tamba	1.68 (1.13 to 2.50)	1.58 (1.04 to 2.39)
Becoming a resident in Tamba	1.60 (0.60 to 4.24)	1.78 (0.69 to 4.59)

Abbreviations: CI, confidence interval; RR, Risk ratio.

* Adjusted for gender, university, and general practice intentions in the final school year.

The effect of experiencing CBME in the senior years was shown to be statistically significant and to persist for several years (1–3 years).

## Discussion

### Major findings

In this study, we found that medical students who had experienced CBME in the senior years were significantly more likely to apply for residency programs in Tamba compared to students who experienced CBME in the junior years or had no experience of CBME. To our knowledge, there have been no studies of short-term CBME programs of one day to two weeks’ duration in which the experience significantly contributed to the workplace a few years later. Although short-term CBME programs of one day to two weeks have been observed to have short-term effects [14–16], this is the first study to confirm the medium-term educational effects of CBME after several years in short-term CBME programs.

### Significance of these findings

The Rural Physician Associate Program [9], University of Missouri Rural Track Pipeline Program [10], and Parallel Rural Community Curriculum [11] are programs that have demonstrated the mid- to long-term educational benefits of CBME. These longer-term CBME programs have been shown to have medium- to long-term effects on actual workplace choice for medical students. Moreover, these programs receive considerable financial support. In such programs, which take place primarily in rural areas, students stay in the region as part of carefully designed longitudinal integrated clerkships for 6–54 weeks. In Japan, by contrast, CBME programs are generally short stays of one day to two weeks [14–16]. Unlike overseas CBME programs, which receive financial support and can be conducted for longer durations, it is difficult to obtain financial support for CBME programs in Japan. Hence, many CBME programs in Japan are limited to short stays.

Surveys conducted immediately after the placement showed a significant increase in medical students’ interest, fulfilment, and enthusiasm for rural medicine in Japan [14–16]. However, no results have been obtained for these short-term CBME programs in Japan and other countries showing medium-term practical training effects on an annual basis. It is important to note that despite the short duration of the CBME program of three days and two nights, the CBME program in Tamba yielded medium-term effects. This could lead to a recruitment drive for medical students to consider CBME programs in rural areas as future workplaces, even in relatively short-term CBME programs where sufficient financial support is not available.

### Notable outcome factors

Our current CBME program has a short duration of three days and two nights, which is consistent with previous studies in Japan. One feature of our CBME program in the Tamba area is the homestay practice with local residents [18]. Host families are openly recruited, and students and residents live together for two days and one night, spending significant time together. This homestay practice is conducted with approximately 10 host families at a time, with 10–15 students with each family simultaneously [18].

Although there have been studies on the effectiveness of medical students’ homestay practice, the simultaneous homestay practice of a large number of students in many host families’ homes is unique and globally unprecedented [22]. Most homestay studies have been conducted overseas (outside of the student’s home country), from the students’ perspectives, with homestay periods ranging from three weeks to two years [22]. This long-term homestay practice has also shown a medium-term practice effect, with trainees working in the homestay location several years later [20]. Further, the homestay training we studied in the Tamba area lasted only one night and two days but still showed short-term training effects [17]. In this study, we also showed a medium-term effect of several years.

CBME has shown that the short-term effect is attachment to the region in which the students practiced and their awareness of the region as a future place of work [17]. This practice effect was shown to persist for at least several years in this study. The homestay practice provides an opportunity for prolonged contact with local residents, a factor that may contribute to the development of healthcare professionals who provide primary care and community medicine in rural areas [18]. We believe that even in this typical one-day to two-week short-term CBME program in Japan, homestay practice, which allows participants to learn about the lives and lifestyles of local residents, will contribute to the establishment of a program that shows the mid-term educational effects of CBME.

### Contribution and next steps

It is significant that this study shows the medium-term educational effects of a short-term CBME program of one day to two weeks, which is the common duration for programs conducted in Japan, in contrast to the long-term CBME programs conducted in other countries. Even if the CBME programs do not receive sufficient financial support, they can have a medium-term effect on the choice of place of work of medical students in their senior years by devising the content of the program. These findings suggest that even short-term CBME programs may show medium-term effects by providing sufficient time for interaction with the local population, particularly through homestays. Outside the Tamba area, which was the focus of this study, CBME programs were offered to medical students in 4–6 other rural areas during the same period. Two avenues for future research are (1) to examine whether similar medium-term effects have been achieved in other rural areas outside Tamba, and (2) to examine the actual program content and the effects (choice of future place of work) obtained from the CBME program.

### Limitations

This study has some limitations. First, the study population was limited to students from the regional quota. Second, the number of training hospitals for which regional-quota students could apply was limited to 10, one of which was Hyogo Prefectural Tamba Medical Center. Third, the final residency program was developed by the relevant department of Hyogo Prefecture while respecting the students’ own applications. Thus, factors other than the will of the students themselves were considered. Consequently, there was no significant difference in the actual initial training sites. Fourth, as stated under the Contribution and Next Steps section, a comparison of applications for residency programs and the content of CBMEs conducted simultaneously in other areas of Hyogo Prefecture after the CBME programs would clarify the characteristics and effects of our program. However, such a comparison was not conducted in this study.

## Conclusions

While our study effectively demonstrates a statistically significant increase in residency program application rates among medical students who participated in the CBME program in the senior years of medical education compared to those who did not, it is important to consider the broader implications of these results. Especially, our findings suggest that short-term CBME programs, even those lasting only three days and two nights, can have medium-term effects on career trajectories, as evidenced by the observed outcomes several years later. This contributes valuable insight into the potential long-lasting benefits of such programs in shaping future medical professionals.

## Data Availability

The datasets used in the study are available from the first author upon reasonable request.
